# Standard Codon Substitution Models Overestimate Purifying Selection for Nonstationary Data

**DOI:** 10.1093/gbe/evw308

**Published:** 2017-01-05

**Authors:** Benjamin D. Kaehler, Von Bing Yap, Gavin A. Huttley

**Affiliations:** 1 Research School of Biology, College of Medicine, Biology, and Environment, Australian National University, Canberra, ACT, Australia; 2 Department of Statistics and Applied Probability, National University of Singapore, Singapore, Singapore

**Keywords:** codon models, natural selection, nonstationary, Markov model

## Abstract

Estimation of natural selection on protein-coding sequences is a key comparative genomics approach for de novo prediction of lineage-specific adaptations. Selective pressure is measured on a per-gene basis by comparing the rate of nonsynonymous substitutions to the rate of synonymous substitutions. All published codon substitution models have been time-reversible and thus assume that sequence composition does not change over time. We previously demonstrated that if time-reversible DNA substitution models are applied in the presence of changing sequence composition, the number of substitutions is systematically biased towards overestimation. We extend these findings to the case of codon substitution models and further demonstrate that the ratio of nonsynonymous to synonymous rates of substitution tends to be underestimated over three data sets of mammals, vertebrates, and insects. Our basis for comparison is a nonstationary codon substitution model that allows sequence composition to change. Goodness-of-fit results demonstrate that our new model tends to fit the data better. Direct measurement of nonstationarity shows that bias in estimates of natural selection and genetic distance increases with the degree of violation of the stationarity assumption. Additionally, inferences drawn under time-reversible models are systematically affected by compositional divergence. As genomic sequences accumulate at an accelerating rate, the importance of accurate *de novo* estimation of natural selection increases. Our results establish that our new model provides a more robust perspective on this fundamental quantity.

## Introduction

Understanding of the action of natural selection on protein-coding sequences underpins fundamental questions regarding evolution and has immediate practical ramifications. It has been known at least because the seminal contribution of King and Jukes (1969) that natural selection for molecular function impacts genetic variation. Natural selection’s influence is now routinely estimated using probabilistic codon models of sequence evolution and reported in genome portals (Hubbard et al. 2009). These models are widely employed for purposes as diverse as seeking to identify genes affecting the natural history of species (Chimpanzee Sequencing and Analysis Consortium 2005) to aiding in the design of new materials (Maitip et al. 2015).

A reasonable model of codon evolution is critical to the measurement of selective pressure. Since the earliest such analysis that corrected for multiple substitutions at a single site (Kimura 1977), it has been assumed that the process of evolution is time-reversible. It is well established that this assumption is incorrect (Karlin and Ladunga 1994). There is also a body of literature where researchers have fitted models of nucleotide evolution that are not time-reversible (Yang and Roberts 1995; Galtier and Gouy 1998; Huelsenbeck et al. 2002; Yap and Speed 2005; Jayaswal et al. 2005, 2007; Baele et al. 2010; Jayaswal et al. 2011, 2014). We recently showed that models of nucleotide substitution that do not assume time-reversibility are statistically feasible in some instances where time-reversible models are not, and that assuming time-reversibility affects inference relating to genetic distance (Kaehler et al. 2015). Regardless, time-reversibility is typically retained as a modeling decision for analysis of biological sequences (nucleic or amino acid) for the sake of convenience.

Differences in codon frequencies between species establish that the process of sequence divergence is nonstationary. For example, the codon AAA is approximately 250 times more abundant in the genome of *Buchnera aphidicola* than that of *Streptomyces venezuelae* (Nakamura et al. 2000). More generally it has been established that codon usage is strongly correlated with genomic GC content (Muto and Osawa 1987). The relationship between nucleotide composition and codon frequency has also been demonstrated to be highly predictive across bacteria, archaea, and eukaryotes (Knight et al. 2001). As argued by Knight et al. (2001), these observations convincingly establish changes to neutral mutation processes as the primary driver for changes in codon frequency. Accordingly, accurate modeling of protein coding sequence evolution requires accommodating these properties of the underlying nucleotide substitution process.

Lindsay et al. (2008) and Yap et al. (2010) showed that the Y98 (Yang 1998) and MG94 (Goldman and Yang 1994; Muse and Gaut 1994) models of codon evolution introduced systematic biases in estimates of natural selection resulting from their construction. Yap et al. (2010) introduced the CNF model to address these shortcomings. The innovation of CNF is in the substitution rate for a single nucleotide substitution in a codon. Instead of using the target codon frequency such as in Y98, the CNF model uses the conditional frequency of the target nucleotide given its unchanged neighboring nucleotides. [The mathematical details are given fully in Yap et al. (2010).] The resulting model produced considerably less-biased estimates of natural selection, as shown in Yap et al. (2010). It is also the best-performing time-reversible model in this study.

Before continuing it is worth clarifying the relationship between nonstationarity and time-reversibility of Markov processes and models. We call a substitution model time-reversible if all of the Markov processes that comprise the model are time-reversible. Similarly, nonstationary models are made up of nonstationary processes. The set of time-reversible models is nested in the set of stationary models, which is in turn nested in the set of nonstationary models. That is to say a time-reversible model cannot be nonstationary, but a nonstationary model can be time-reversible for certain choices of parameters. We define nonstationarity properly in the “Materials and Methods” section.

Care is required when fitting nonstationary models to ensure that they can be *consistently* estimated. In this context, consistency means that if a multiple sequence alignment was generated under a particular model and we fit the same class of model to the alignment, then the estimates should tend toward the generating parameters as the length of the alignment increases. We use the results of Chang (1996) to ensure that our models can be consistently estimated, as this is a key feature of probabilistic methods that distinguish them from earlier methods in phylogenetics (Felsenstein 1978).

In this paper, we extend the techniques for calculating genetic distance and establishing consistency for nonstationary models developed in Kaehler et al. (2015) to the context of codon substitution models, and reveal that the assumption of time-reversibility in codon substitution models introduces systematic bias. Some authors have considered amino acid models without the assumption of time-reversibility (Groussin et al. 2013; Parks 2014), but to the best of our knowledge, this is the first example of a codon model that does not impose time-reversibility to allow direct estimation of natural selection.

Natural selection is frequently estimated using the parameter *ω*, which allows the rate of synonymous and nonsynonymous codon substitutions to vary in a way that is not consistent with neutral evolution. Under a given model of neutral evolution, *ω* is defined so that *ω* = 1 represents neutral evolution, ω>1 positive Darwinian selection, and ω<1 purifying selection. This quantity has a complicated history, with *ω* having also been called *dN*/*dS* (Nei and Gojobori 1986) and $K_{a}/K_{s}$ (Miyata and Yasunaga 1980). Prior to the dominance of maximum-likelihood (subsequently Bayesian) methods, several empirical methods were devised for its calculation. Early contributors to the field identified the need for simpler approaches (Kimura 1980; Nei and Gojobori 1986).

Equivalently, *ω* enters the parameterization of a continuous-time Markov model of codon substitution as a multiplicative constant for all nonsynonymous codon transitions (Goldman and Yang 1994; Muse and Gaut 1994) which is then fitted using maximum-likelihood or Bayesian techniques. The specifications for these models have developed over time but we focus on two choices. The first is that of Yang (1998), which we shall call Y98. This model is described in detail below but for the moment we will describe it as an extension of the nucleotide model of Hasegawa et al. (1985). It is currently the most widely used model of codon substitution. The second is one of those developed in Yap et al. (2010), which we shall denote CNFGTR, for conditional nucleotide frequency general time reversible. Y98, CNFGTR, and all previously published codon substitution models are time-reversible.

CNFGTR is the CNF extension of the general time reversible (GTR) nucleotide substitution model (Lanave et al. 1984) that addresses a bias present in Y98 when nucleotides are not equiprobable. In Yap et al. (2010) it was shown that CNFGTR most effectively reduced that bias in Y98 and other biases shown to be present in MG94.

In Kaehler et al. (2015) it was observed using parametric bootstraps (Goldman 1993) that for alignments of third codon position nucleotides from nuclear encoded genes in a triad of mammals, mitochondrial protein coding genes from the same mammals, and ribosomal RNA from microbes that time-reversible models never feasibly described the process that generated the data. A more general, nonstationary model could feasibly have generated the mammal nuclear encoded gene data set, and succeeded more often as a reasonable model for the mtDNA and rRNA data sets. It was also shown that the time-reversible models systematically overestimated genetic distance in a manner proportional to a nonparametric measure of nonstationarity. Further, departure from the molecular clock hypothesis was overstated by time-reversible models, demonstrating that biological inferences based on estimates of the number of substitutions were affected by use of the time-reversible models. This work required new theoretical insights into calculation of genetic distance in the nonstationary setting.

As an alternative to time-reversible codon substitution models we present the nonstationary GNC or general nucleotide codon model, which extends the nonstationary nucleotide model presented in Kaehler et al. (2015). We extend the theoretical insights in Kaehler et al. (2015) in this setting and show that it can be used to estimate *ω* in a manner consistent with its forebears. We test large sets of alignments of mammals (human, mouse, and opossum), vertebrates (human, xenopus, and fugu), and ants (Florida carpenter ant, Indian jumping ant, and Argentine ant) to show that GNC tends to fit the available data better than time-reversible models. We further demonstrate that consistent with Kaehler et al. (2015), Y98 and CNFGTR tend to overestimate genetic distance in comparison with GNC in a manner proportional to a nonparametric measure of nonstationarity and link overestimation of genetic distance with underestimation of *ω*. Similar to Yap et al. (2010), we use intronic data to show that Y98 estimates *ω* with a bias that is a function of sequence GC content, but that CNFGTR and GNC are not biased in this way. We also demonstrate that inference regarding the molecular clock hypothesis is affected by model time-reversibility.

The “Materials and Methods” section specifies GNC, the time-reversible models against which we compare GNC, model fitting techniques and theoretical considerations, and the data sets on which we test our methods. The “Results” section details our simulation and empirical findings, which are interpreted in the “Discussion” section.

## Materials and Methods

### The General Nucleotide Codon Model

The general nucleotide codon (GNC) model is a nonstationary, continuous-time Markov process. For time-reversible models, the location of the root of the phylogenetic tree has no bearing on the model’s predictions. For nonstationary models, the location of the root matters as the process evolves forward in time away from the root.

At the root node of the tree, each codon is assigned an initial probability with no further assumptions, so forming a 61-element row vector *π^r^*. In this notation we have labeled the root node *r* and specified *π^r^* at the root node because the codon composition can change through time and between nodes. The conditional nucleotide processes for each codon position are identical except that any rate corresponding to a nucleotide substitution that results in a nonsynonymous codon substitution is multiplied by the parameter *ω*, with no further constraints. We show in the Appendix that *ω* is equal to $K_{a}/K_{s}$ as defined in, for example, Goldman and Yang (1994).

Equivalently, we write that the codon process on an edge joining nodes *a* and *b*, or {*a*, *b*}, is defined by the Markov generator *Q^ab^* with off-diagonal elements labeled by codons $i_{1}i_{2}i_{3}$ and $j_{1}j_{2}j_{3}$:

(1) $$q_{i_{1}i_{2}i_{3},j_{1}j_{2}j_{3}}^{ab}=\{\begin{array}{cc} 0, & \text{more}\;\text{than}\;\text{one}\;i_{n}\neq j_{n},\\ r_{i_{n}j_{n}}^{ab}, & i_{n}\neq j_{n},\;\;\text{synonymous}\;,\\ \omega^{ab}r_{i_{n}j_{n}}^{ab}, & i_{n}\neq j_{n},\;\;\text{nonsynonymous}\;, \end{array}$$

where n∈{1,2,3}}, *ω^ab^* is the selective pressure on the edge {*a*, *b*}, and *r^ab^* is in turn a matrix that defines the neutral nucleotide process. It is important to note that the node *b* is further from the root node than *a*, which is the concrete consequence of each edge having a direction in time. Diagonal elements of *Q^ab^* are calculated to satisfy the constraint that row sums must be zero. We introduce a scale parameter *τ^ab^* and scale *Q^ab^* such that $-\pi^{r}\cdot \text{diag}Q^{ab}=1$. The transition probability matrix on {*a*, *b*} is then $P^{ab}=\;\text{exp}\;\left\{Q^{ab}\tau^{ab}\right\}$.

Note that *π^r^* is absent from (1). This is in contrast to the time-reversible models, (3) and (4), where the codon probabilities are included in such a way as to force the process to be time-reversible.

We digress briefly to outline the relationship between the scale parameter *τ*, time and genetic distance. Recall that genetic distance is measured as the expected number of substitutions between two nodes in the tree. Further, genetic distance is typically employed as a measure of “evolutionary time”. For time-reversible models, the expected number of substitutions along one edge equals the scale parameter. Accordingly, where we refer to a scale parameter in the context of a time-reversible model, it can safely be interpreted as the time parameter.

For nonstationary models, such as GNC, *τ* does not necessarily equal the expected number of substitutions (Kaehler et al. 2015). Rather, the genetic distance can be calculated as

(2) $$d_{\text{GNC}}^{ab}=-\pi^{a}\int_{0}^{\tau^{ab}}\;\text{exp}\;\left\{Q^{ab}s\right\}ds\;\text{diag}\;\;Q^{ab}.$$

for the edge {*a*, *b*} where *π^a^* is the row vector of codon probabilities at node *a*. The derivation of this formula carries without modification from Kaehler et al. (2015). Genetic distance across multiple edges is the sum of the distances for those edges. Numerical methods for calculation of the integral of the matrix exponential are discussed in Kaehler et al. (2015).

### Fitting Nonstationary Models

As stated in the “Introduction” section, almost all nucleotide and all codon models in common use are time-reversible, so stationary. We therefore use this section to review some fundamental results regarding nonstationary models and clarify how they can be used in practice.

Stationarity is a property of a Markov process. A Markov process is defined by the probabilities of transitions between states between points in time and an initial probability distribution. In our current context, the states are the 61 sense codons. Under very mild assumptions, a Markov process has a unique stationary distribution that is determined by the transition probabilities. It is the distribution into which the process will settle if allowed to run for enough time. If the initial distribution is equal to the stationary distribution, the distribution does not change through time and we call the process stationary. If the initial distribution is any other probability distribution, the process is nonstationary. For more detail in a phylogenetic context see Liò and Goldman (1998). For more depth, there are several good introductory texts on Markov processes (Hoel et al. 1986).

A phylogenetic model is made up of a phylogenetic tree and a Markov process associated with every edge in the tree. It is common to assume that every edge has the same process and that the initial distribution at the root of the tree is the stationary distribution for that process, so that every process on the tree is stationary. It is usually also assumed that the processes are time-reversible, which would not be possible if they were not stationary. It is possible to define a phylogenetic model that has a different process on every edge that is still stationary, because different Markov processes can share the same stationary distribution. This is in fact common in the application of codon models to estimate whether selective pressure varies between edges of a phylogeny, and is true of all of the time-reversible models that are tested in this paper, where selective pressure is allowed to vary by edge.

GNC is nonstationary because the initial distribution is not the stationary distribution of the process on each edge. That means that codon composition is allowed to vary along edges of the tree. It does not automatically mean that every edge on the tree has a different process. We could assign the same process to every edge of the tree and it would still be nonstationary. This topic is treated in greater depth in Jermiin et al. (2008).

We now turn our attention to the concrete implications for the statistics that we calculate in this work.

**Fig. 1.—:**
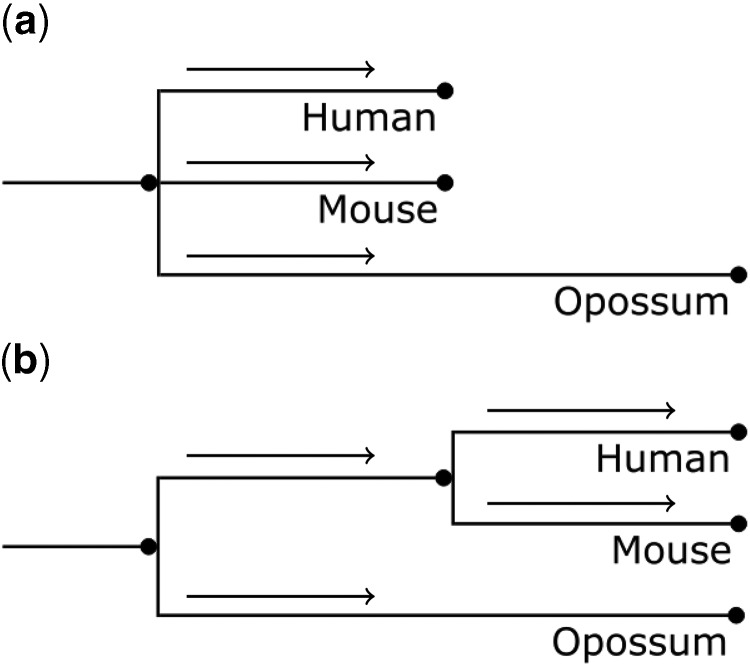
Tree topologies; (*a*) node-rooted, (*b*) edge-rooted. The stems illustrate the placement of the roots, but do not form part of the model. Arrows indicate the direction of time.

Recall that the *degree* of a node is the number of edges that connect to that node. In this work, we will make the distinction between trees that are node-rooted (fig. 1*a*), where the root node has degree three, and edge-rooted (fig. 1*b*), where the root node has degree two. If we assume that the process on each edge of the tree is time-reversible, then we would ordinarily call a node-rooted tree *unrooted*, because the direction of time on each edge is irrelevant. In a nonstationary context, the direction of time matters and is decided by the position of the root, so we prefer the term node-rooted to the term unrooted. The direction of time is illustrated in figure 1 for varying root positions.

We reiterate that it is not possible to consistently fit a general Markov model on an edge-rooted topology to data from a multiple sequence alignment. Further, it is only possible to fit a general Markov model on a topology with three or more taxa. These results are explored in depth in Chang (1996). They follow from the fact that a general Markov model is not identifiable for an edge-rooted topology with two taxa. That is, in many such cases, there are multiple sets of parameters that give rise to exactly the same observable alignment column frequencies. For certain parameter choices, the same is true for GNC. It is possible that sufficient additional assumptions could be placed on GNC to make it identifiable for an edge-rooted tree. Proof that GNC is identifiable on an edge-rooted tree would be a ground-breaking contribution to the field of phylogenetics, but such a proof does not exist. We must therefore assume that GNC must be fitted to a node-rooted tree with at least three taxa. We make an exception to this rule for the simulation studies where we do not claim to fit an identifiable model.

Once we have dropped the assumptions of time-reversibility and stationarity, it is natural to ask whether every edge on the tree should be associated with a different Markov process. The answer is that it depends on the application. Allowing every edge to have its own process quickly increases the number of parameters in the model which reduces computational tractability and increases susceptibility to overfitting. In the simplest case, we could associate a single model with every edge of the tree, as in the work of Yap and Speed (2005). If we suspect that different models should be associated with different parts of the tree, algorithms exist for automatically determining where to add this complexity (Jayaswal et al. 2011, 2014). It is also possible to allow some parameters to vary among edges (Galtier and Gouy 1998; Groussin et al. 2013), which is a likely scenario where we wish to test hypotheses about selective pressure.

It is possible to use GNC to discover a tree topology, and under a Bayesian framework it would be natural to incorporate uncertainty about the topology. Ordinarily, however, it would be unusual to use any codon model for this purpose, as they are generally slower to fit due to the computational demands of exponentiating larger matrices.

For this work, we are concerned with discovering the properties of evolutionary processes, not the discovery of tree topologies. We therefore choose the smallest number of taxa that we can use for this purpose and exclusively fit our models to sets of three taxa. Using a three-taxon node-rooted tree also has the advantage of removing uncertainty regarding the tree topology as there is exactly one such tree. Further, as the number of taxa is small, we always allow the GNC process to be different on each of the three edges. Again, we make an exception to this rule for the simulation studies. In some instances we will make some parameters common across some edges while allowing others to vary. Unless we state otherwise all models are fitted to node-rooted trees.

### Checks for Consistency

Application of Chang (1996) to show that GNC can be consistently estimated requires care due to two technicalities. These issues were encountered in Kaehler et al. (2015), and we overcome them in the same fashion here.

First, states at internal nodes can be relabeled without affecting alignment column frequencies. This issue is explained in detail in Zou et al. (2011) for the Barry and Hartigan (1987) model, but is applicable here by Remark 2 in Chang (1996). The issue can be resolved through imposing the constraint suggested by Remark 9 in Chang (1996), which is that the fitted transition probability matrices be Diagonal Largest in Column (DLC).

Second, multiple continuous-time processes can have the same alignment column frequencies as a single discrete-time process (Higham 2008). That is, it is possible for valid transition rate matrices *Q*_1_ and *Q*_2_, where $Q_{1}\neq Q_{2}$, to give rise to the same transition probability matrix so that $e^{Q_{1}}=e^{Q_{2}}$. In fact, one transition probability matrix can correspond to zero, one, several, or a continuum of transition rate matrices. It is possible to conservatively identify cases where more than one valid transition rate matrix exists using Theorem 1.27 in Higham (2008). We call any such case a nonunique mapping. We note that a stricter sufficient condition for uniqueness was necessary for GNC than was employed in Kaehler et al. (2015), as the problem is substantially harder for a 61 × 61 matrix than it is for a 4 × 4 matrix.

### Model Implementation

All substitution models were fitted using PyCogent (Knight et al. 2007). GNC has not yet been incorporated into the PyCogent library, but is available with the code used to execute these analyses at https://bitbucket.org/nonstationary/codon (last accessed January 9, 2017). All simulations were also performed using PyCogent.

As stated above, Y98 is the codon model specified in Yang (1998). In this model, a 61-element stationary distribution *π* is defined on the sense codons. The Markov generator, labeled by codons *i* and *j*, has off-diagonal elements

(3) $$q_{ij}=\{\begin{array}{cc} \pi_{j}, & \;\text{one}\;\text{synonymous}\;\text{transversion}\;,\\ \kappa \pi_{j}, & \;\text{one}\;\text{synonymous}\;\text{transition}\;,\\ \omega \pi_{j}, & \;\text{one}\;\text{nonsynonymous}\;\text{transversion}\;,\\ \omega \kappa \pi_{j}, & \;\text{one}\;\text{nonsynonymous}\;\text{transition}\;,\\ 0, & \;\text{otherwise}\;. \end{array}$$

Y98 is time-reversible.

The CNFGTR model is defined in Yap et al. (2010). It also has a 61-element stationary distribution. Its Markov generator’s off-diagonal elements, labeled by codons $i_{1}i_{2}i_{3}$ and $j_{1}j_{2}j_{3}$, is given by

(4) $$q_{i_{1}i_{2}i_{3},j_{1}j_{2}j_{3}}=\{\begin{array}{cc} 0, & \;\text{more}\;\text{than}\;\text{one}\;i_{n}\neq j_{n},\\ r_{i_{n},j_{n}}\pi_{j_{n}|{\left\{j_{m}\}\right\}}_{m\neq n}}, & i_{n}\neq j_{n},\;\text{synonymous}\;,\\ \omega r_{i_{n},j_{n}}\pi_{j_{n}|{\left\{j_{m}\}\right\}}_{m\neq n}}, & i_{n}\neq j_{n},\;\text{nonsynonymous}\;, \end{array}$$

where $n,m\in \{1,2,3\}\},\;r_{i,j}$ are the elements of a 4 × 4 symmetric matrix, and $\pi_{j_{n}|{\left\{j_{m}\}\right\}}_{m\neq n}}$ is the probability of observing *j_n_* given the other two codon positions *j_m_*. CNFGTR is time-reversible.

The formulation of the above models and GNC with 61 states is, of course, dependent on the genetic code associated with the alignments to which the models are fitted. The software that we provide is agnostic to genetic code and is easily configurable for alternatives, as we did for tests on intronic data where we allowed stop codons.

In every case, *ω* represents the influence of natural selection, which is sometimes also referred to as *dN*/*dS* (Yap et al. 2010) or $K_{a}/K_{s}$ (Goldman and Yang 1994).

For the time-reversible models, the scale parameter *τ* and selective pressure *ω* were allowed to vary by edge, except where otherwise noted. For time-reversible and nonstationary models, codon probabilities were fitted along with the other parameters.

In this standard scenario, the initial distribution contributes 60 parameters to each model. For Y98, *κ* contributes one additional parameter and *τ* and *ω* contribute two parameters per edge, making a total of 67 parameters for a three-taxon tree. For CNFGTR, the GTR parameters add five parameters to the model and *τ* and *ω* again add two parameters per edge, summing to 71 parameters. GNC is most parameter-rich, with 12 rate parameters plus *ω* per edge for 99 parameters overall.

Genetic distances were calculated as the expected number of substitutions per codon. The expected number of substitutions for nonstationary models was calculated following the method of Kaehler et al. (2015), as noted above.

### Assessment of Model Goodness-of-Fit

For convenience, we here reproduce from Kaehler et al. (2015) our description of how parametric bootstraps (Goldman 1993) and the G-statistic (Sokal and Rohlf 1995, pp. 686–697) were used to calculate *P*-values that objectively reflect goodness of fit for a particular model. The null hypothesis says that the alignment is generated by the fitted model with parameter values set at our estimates. The expected site-pattern counts under the model are thus the probability of the pattern multiplied by the alignment length. The alternative is the unrestricted multinomial model, as described in Goldman (1993), taken as the observed site-pattern counts in the alignment. The G-statistic is computed from the expected and observed counts using the conventional expression (Goldman 1993, pp. 686–697). The bootstrap procedure is to

1. simulate 49 alignments of the same length as the fitted alignment under the null hypothesis;
2. perform the original fit on each simulated alignment; and
3. calculate the proportion of fitted G statistics that exceed or equal that of the original statistic.

The result is the G-statistic parametric bootstrap *P*-value.

### Data

We analyzed four sets of data. Each was made up of thousands of multiple sequence alignments of orthologous sequences from three taxa. The taxa are common across every alignment in a given data set. We label the data sets as mammal, vertebrate, ant, and intronic. This section details the contents of each data set, how they were obtained, and any filtering that was applied.

We obtained the mammal and vertebrate data sets by downloading protein coding sequences from Ensembl 68. Each multiple sequence alignment in the mammal data set was made up of three one-to-one orthologs from *Homo sapiens, Mus musculus*, and *Monodelphis domestica* and each alignment in the vertebrate data set consisted of three one-to-one orthologs from *Homo sapiens, Takifugu rubripes*, and *Xenopus tropicalis*. PyCogent (Knight et al. 2007) was used to download these sequences.

Alignments of intronic one-to-one orthologous sequences for *Homo sapiens, Macaca mulatta*, and *Callithrix jacchus* were downloaded from Ensembl 81. Regions annotated in Ensembl as low complexity were masked and those columns removed from the alignments in a manner that preserved naturally occurring trinucleotides. Again, PyCogent was used to obtain the data.

Another set of protein coding sequences were downloaded from the Ant Genome Portal (Munoz-Torres et al. 2011) for *Camponotus floridanus, Harpegnathos saltator*, and *Linepithema humile*. One-to-one orthologous sequences were selected using three-way reciprocal BLAST (Camacho et al. 2009), meaning that for each alignment, every sequence was the top BLAST hit for the other two sequences.

All protein coding sequences were aligned using PyCogent assuming a CNFHKY model (Yap et al. 2010).

In every instance, any column in a multiple sequence alignment that contained a non-nucleotide character was excluded and only alignments with at least 500 remaining codons were analyzed. All data sets are available on Zenodo at http://doi.org/10.5281/zenodo.192513 (last accessed January 9, 2017).

Any alignments that resulted in GNC fits that did not satisfy consistency checks (DLC and unique mapping constraints) were excluded. The mammal, vertebrate, and ant data sets lost 17, 879, and 1 alignments to the DLC constraint respectively and the mammal, vertebrate, ant, and intronic data sets lost 94, 20, 10, and 2,139 alignments to the strict unique mapping constraint, respectively. These numbers are interesting in themselves as they relate to the limits of inference for GNC, but we observed that removing these data points made no material difference to our results.

The mammal, vertebrate, ant, and intronic data sets ultimately contained 4,039, 2,008, 2,008, and 10,907 alignments, respectively. The median length of the alignments in nucleotides was 2,256, 2,376, 2,257.5, and 10,113 for each data set in the same order.

## Results

### Simulation Results

Our goal in this work is to assess how nonstationarity in the observed data affects inference drawn using time-reversible models, specifically regarding questions of selective pressure and genetic distance. In particular, we will compare GNC with Y98 from Yang (1998) and CNFGTR from Yap et al. (2010). GNC is nonstationary, whereas CNFGTR and Y98 are time-reversible.

It is necessary to fit GNC to a node-rooted tree, but in reality we would expect a phylogenetic tree to be edge-rooted. We therefore fitted GNC on an edge-rooted tree to nine alignments from the mammal data set, in the knowledge that the fitted model was not consistently estimable, then simulated 100 alignments from each fitted model. We then evaluated whether GNC on node-rooted trees could serve as a reliable proxy for the true nonstationary process under which the observed sequences were simulated, at least for the purpose of comparison with results from CNFGTR and Y98, by fitting all three models to each simulated alignment.

The chosen alignments were of one-to-one orthologs of the Human genes ENSG00000005436, ENSG00000024526, ENSG00000106443, ENSG00000125207, ENSG00000139517, ENSG00000162614, ENSG00000176371, ENSG00000180488, and ENSG00000239305. The alignments were selected to maximize the observed difference in codon composition between the Human and Mouse sequences. Difference in composition was measured using Jensen–Shannon divergence (JSD) (Lin 1991). As we were concerned about the identifiability of GNC on an edge-rooted tree, we set all parameters excluding the scaling parameter to be equal on the Opossum edge and the internal edge. Otherwise all parameters were allowed to vary by edge. We note that the goal here was not to fit a model for the purpose of inference, but rather to create a known, biologically plausible, edge-rooted model for the purpose of simulation.

**Fig. 2.—:**
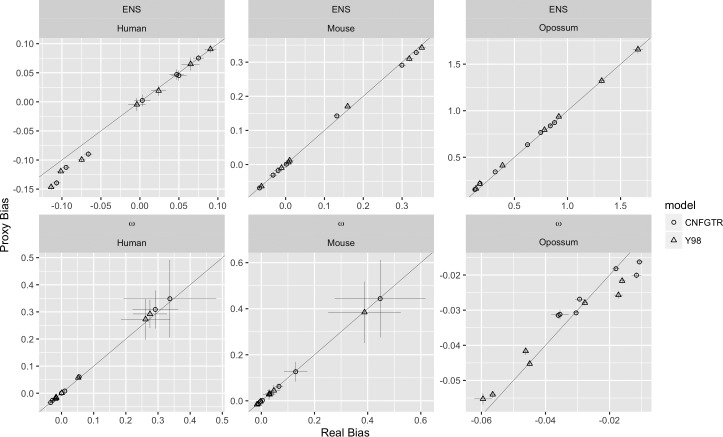
Effect of using GNC with a node-rooted tree to estimate bias in CNFGTR and Y98 for nine alignments generated using GNC with an edge-rooted tree. Proxy bias is the average difference between the time-reversible model estimate and the node-rooted GNC estimate over up to 100 simulations. True bias is the average difference between the time-reversible model estimates and the known parameters. Time-reversible model fits of simulated alignments were excluded if ω>10, so 46 out of 900 fits were excluded. The feint diagonal line shows the diagonal and cross-hairs show 95% CIs.

We are interested in bias in estimated genetic distance and selective pressure. True bias was directly estimated as the generating model was known, and proxy bias was calculated by subtracting the GNC estimates of the parameters from the estimates under the time-reversible models. The results are shown in figure 2.

We see that the bias introduced by fitting time-reversible models to data generated by a nonstationary model exceeds the bias introduced by fitting a model on a node-rooted tree as most points fall close to the diagonal. We also note that these results show that our numerical model fitting procedure is capable of reconstructing the parameters of the generating models, at least for genetic distance and selective pressure.

There are two small artifacts in figure 2, where the genetic distance bias is negative on the Human edge, and for selective pressure on the Opossum edge. We shall see that we are largely interested in overestimation, that is positive bias, of time-reversible models for genetic distance, so the small difference between the proxy bias and the true bias on the Human edge are not material to our results. For the Opossum selective pressure results, we note that both proxy and true bias are close to zero.

The simulation results show that for a small selection of alignments that comparing CNFGTR and Y98 with GNC on a node-rooted tree gives reasonable estimates of the bias introduced by fitting CNFGTR and Y98 to data produced by GNC on an edge-rooted tree. What those biases tend to be in nature is addressed in the following sections.

### The Nonstationary Model Fits Better than the Time-Reversible Models

**Fig. 3.—:**
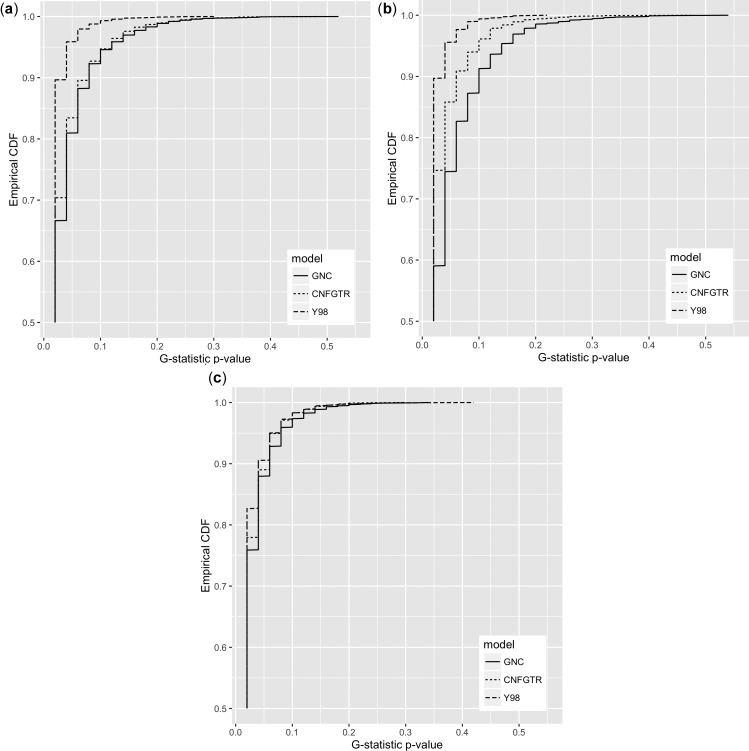
Empirical cumulative distribution functions of *P*-values for the null hypothesis that the alignment could have been generated by the fitted model. Hypotheses were tested using the G-statistic and 49 parametric bootstrap iterations. Datasets consisted of (*a*) 4,039 alignments of mammal protein-coding sequences, (*b*) 2,008 protein-coding ant alignments, and (*c*) 2,008 protein-coding alignments of vertebrates.

For GNC, CNFGTR, and Y98 and the mammal, vertebrate, and ant data sets, we tested the null hypothesis that the data were generated by the fitted model against the alternative hypothesis that they were not. We used 49 bootstrap iterations for every test. This number is low for this type of test, so it is worth mentioning that these calculations were particularly computationally intensive, requiring the equivalent of well over 100,000 h of computation on a single computing core. Table 1 shows the proportion of alignments in each data set that rejected the null hypothesis at nominal 5% significance. While none of the models or data sets reproduced the results in Kaehler et al. (2015), where rejection rates were as low as 6%, GNC consistently performed better than CNFGTR, which in turn outperformed Y98. The full distributions of the p-values for these tests are shown in figure 3.

**Table 1:** ****GNC Dominates Goodness-of-Fit Tests; CNFGTR Fairs Better than Y98

	GNC	CNFGTR	Y98
Mammals	81.0%	83.4%	95.9%
Vertebrates	87.9%	89.0%	90.5%
Ants	74.5%	85.8%	95.6%

### Time-Reversible Models Overestimate Genetic Distance in a Manner Proportional to Observed Nonstationarity

It was observed in Kaehler et al. (2015) that time-reversible nucleotide substitution models tend to systematically overestimate genetic distance in comparison with a nonstationary model, in a manner proportional to observed nonstationarity. We report the same phenomenon for codon substitution models. We fitted GNC, CNFGTR, and Y98 to the mammal, vertebrate, and ant data sets.

**Fig. 4.—:**
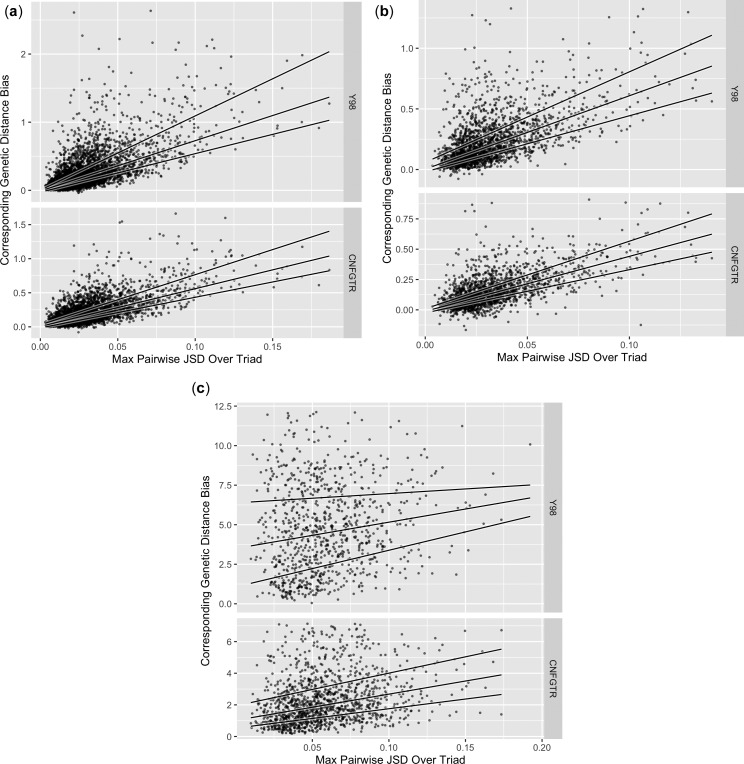
The genetic distance bias increases with Jensen–Shannon Divergence (JSD). Genetic distance biases are $d_{Y98}-d_{\text{GNC}}$ or $d_{\text{CNFGTR}}-d_{\text{GNC}}$, where *d* is the genetic distance under the model indicated by the subscript. Genetic distance is the expected number of substitutions. Measurement was between the pair of taxa with maximal JSD for a given alignment. In every case, the time-reversible models tend overwhelmingly towards overestimation. Solid lines show quantile regressions for 25%, 50%, and 75% quantiles. (*a*) shows 4,030 (Y98) and 4,038 (CNFGTR) alignments of Human, Mouse, and Opossum protein coding genes. (*b*) shows 2,008 alignments of Florida carpenter Ant, Argentine Ant, and Indian Jumping Ant. (*c*) shows 899 (Y98) and 1,340 (CNFGTR) alignments of Human, Xenopus, and Fugu. Plots have been cropped to remove outliers.

Define $d_{Y98}$ and $d_{\text{CNFGTR}}$ as the expected number of substitutions over a path through a tree as inferred from the Y98 and CNFGTR models respectively. $d_{\text{GNC}}$ is defined above in (2). As justified by our simulation and model fit results, we quantified the genetic distance bias of time-reversible models using $d_{Y98}-d_{\text{GNC}}$ and $d_{\text{CNFGTR}}-d_{\text{GNC}}$. For a pair of sequences, we take conservation of codon composition as a measure of stationarity. We use the Jensen-Shannon divergence (JSD) (Lin 1991) for this purpose. Figure 4 shows the relationship between JSD and genetic distance bias for three data sets. Each point in those scatter plots represents a single alignment, where the JSD and genetic distance are taken for the pair of taxa with maximal JSD within the triad. Alignments were excluded from the analysis if their scale parameter on any edge attained a preset maximum value (of 10). No alignment from the ant dataset was eliminated in this fashion for any model. For CNFGTR and Y98, one and nine alignments respectively were eliminated from the mammal data set. For CNFGTR and Y98, 668 and 1,109 alignments, respectively, were excluded from the vertebrate data set. None were excluded from the vertebrate data set for GNC, but the data had already been filtered using the DLC criterion that removed any such examples. We performed quartile regressions of genetic distance bias against JSD for each data set. For CNFGTR, the regression slopes were 5.5, 16.6, and 4.5 to one decimal place for the mammals, vertebrates, and ants, respectively. For Y98, the slopes were 7.4, 16.6, and 6.1 to the same precision in the same order. To one decimal place, the regression intercepts were zero for both models for the ant and mammal datasets. For the vertebrates, the intercepts were 1.0 and 3.5 to one decimal place for CNFGTR and Y98, respectively.

The overwhelming observation is that genetic distance bias tends to be positive, and increases with increasing nonstationarity. For the mammal and ant data sets, Y98 yields genetic distance biases that grow more quickly with increasing JSD than those for CNFGTR. For these data sets, the intercepts are close to zero. For the vertebrate data set, the same trend is present but less clear. We observe that for this data set the fitted scale parameters for the time-reversible models were often at the preset bound of 10, indicating that to these models the number of substitutions often looks saturated. We speculate that this is a source of noise that obscures these results, even if the edge lengths are not completely saturated.

### Time-Reversible Models Underestimate *ω* in a Manner Proportional to Their Overestimation of Genetic Distance

A key feature of a codon model is its ability to estimate natural selection. We will show that the parameter representing natural selection, *ω*, as estimated using time-reversible models, is systematically biased in comparison to its measurement using the nonstationary model. For this purpose, we fitted GNC, CNFGTR, and Y98 to the mammal, vertebrate, and ant data sets.

**Fig. 5.—:**
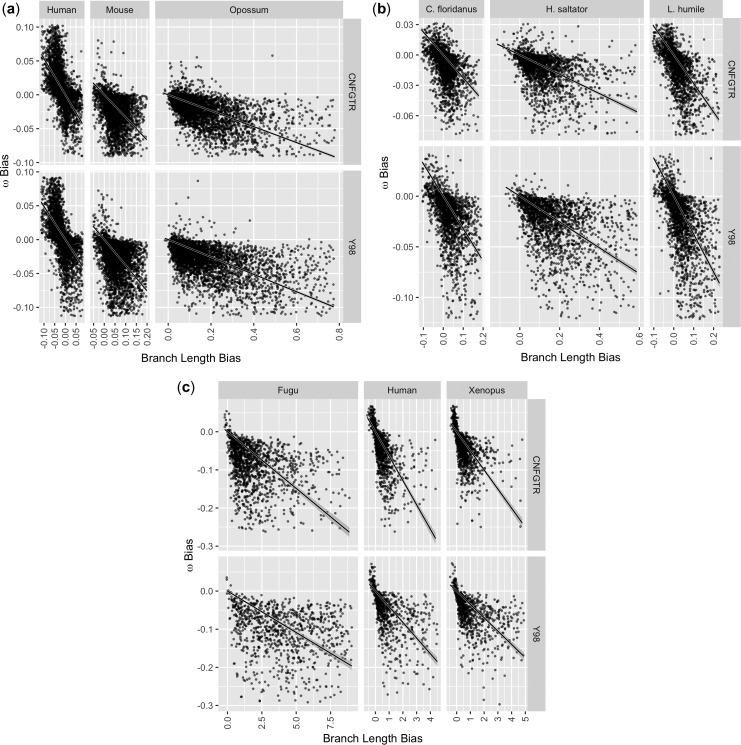
*ω* bias is negatively correlated with genetic distance bias. An empirical relationship exists between genetic distance bias and *ω* bias. In every case, the overestimation of genetic distance is linked to underestimation of *ω*. Solid lines show linear regressions through the origin. (*a*) shows 4,030 (Y98) and 4,038 (CNFGTR) alignments of Human, Mouse, and Opossum protein coding genes. (*b*) shows 2,008 alignments of Florida carpenter Ant, Argentine Ant, and Indian Jumping Ant. (*c*) shows 899 (Y98) and 1,340 (CNFGTR) alignments of Human, Xenopus, and Fugu. Plots have been cropped to remove outliers.

We expect that if edge length is overestimated, then the corresponding parameter *ω* should be underestimated to compensate. We reason that to obtain an observed level of amino acid conservation, if the genetic distance is greater, then the damping effect of *ω* must also be greater. As we have estimated edge length and *ω* separately for each edge we can compare genetic distance bias and *ω* bias directly, as we do in figure 5. Genetic distance bias is calculated as described in the last section and *ω* bias is calculated analogously as $\omega_{\text{GNC}}-\omega_{Y98}$ and $\omega_{\text{GNC}}-\omega_{\text{CNFGTR}}$. Again, where the edge scale parameter attained its maximum allowed value for any edge in an alignment, that alignment was excluded from the analysis; see the previous section for details.

We observed a strong negative correlation between genetic distance bias and *ω* bias. As lower *ω* means that nonsynonymous substitutions are more harshly penalized relative to synonymous substitutions, then underestimating *ω* corresponds to overestimating purifying natural selection. While the relationship between genetic distance bias and *ω* bias is noisy, we note that the majority of the mass for each scatter plot in figure 5 falls in the second and fourth quadrants. For this reason, we do not speculate on the form of the functional relationship, but draw regressions through the origin for each plot.

### Biases That Affect Time-Reversible Models Do Not Affect a Simple Non-stationary Model

Yap et al. (2010) used experiments on intronic regions as controls to test for bias in estimates of *ω* in the time-reversible codon models that they considered. This method has great appeal because it allows us to test our models of natural selection on naturally occurring sequences that we expect to have evolved under the neutral process for protein coding content, and as such constitute a negative control for which we would expect *ω* = 1. The models used for these experiments had to be modified slightly to allow stop codons in a similar fashion to Yap et al. (2010).

**Fig. 6.—:**
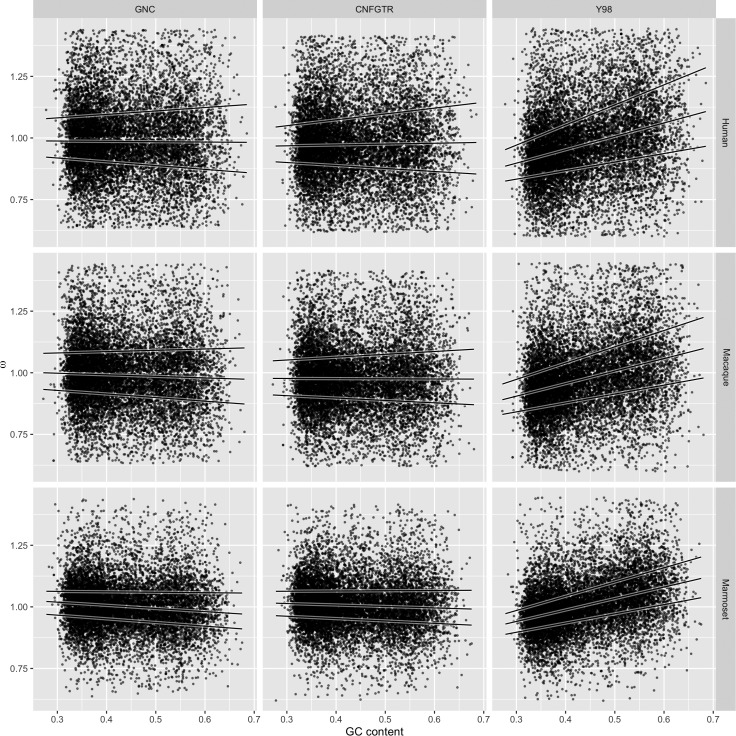
*ω* is systematically biased when estimated using Y98 and CNFGTR, but not GNC. *ω*  = 1 is expected for these 10,907 intronic alignments of human, macaque, and marmoset. Scatter plots relate GC content to estimates of *ω*. Solid lines show quantile regressions for 25%, 50%, and 75% quantiles.

As Yap et al. (2010) compared estimates of *ω* with the measured GC content of the sequences, we do the same in figure 6, with details in table 2. In each case, we fitted GNC, CNFGTR, and Y98 to the intronic data set.

**Table 2:** **** *ω* Estimation Is Strongly Biased when using Y98, Weakly Biased when using CNFGTR, and Least Biased using GNC

	Median *ω*			Median regression slope		
	GNC	CNFGTR	Y98	GNC	CNFGTR	Y98
Marmoset	1.005	1.006	0.998	−0.128	−0.060	0.465
Macaque	0.990	0.976	0.970	−0.064	−0.007	0.508
Human	0.986	0.973	0.965	−0.014	0.035	0.544

We verify the finding in Yap et al. (2010) that $\omega_{Y98}$ has a strong bias that varies with GC content. Lower GC content biases $\omega_{Y98}$ toward underestimation and higher GC content biases $\omega_{Y98}$ toward overestimation. Overall, median $\omega_{Y98}$ is consistently less than one. It is interesting to note that of the three edges, the median estimate of *ω* is close to one for all three models for the marmoset edge. The true root of the evolutionary tree for these species is expected to lie on this edge, so we speculate that this is a manifestation of a different underlying process operating on this edge.

The key finding for our tests on intronic data is that *ω* bias does not appear to vary by GC content for GNC or CNFGTR, with the slopes for the median regression lines for these models being close to zero. By inspecting the median $\omega_{\text{CNFGTR}}$ we see that it again slightly underestimates *ω* for the ingroup edges. This effect is not pronounced for the GNC results.

That Y98 produces *ω* estimates that are biased when the nucleotides are not equiprobable is a consequence of the presence of codon probability parameters in the Markov generator, so that substitution rates are confounded with codon probability parameters. A full description of this phenomenon is given in Lindsay et al. (2008). Models such as that of Muse and Gaut (1994), where the transition rates are multiplied by nucleotide frequencies rather than codon frequencies are less susceptible to this type of bias, and this property is extended by design to conditional nucleotide frequency models such as CNFGTR (Yap et al. 2010). The Muse and Gaut (1994) model has other biases that are introduced because codon probabilities do not appear to be decomposable into nucleotide probabilities, which are also addressed by CNFGTR. GNC is not exposed to bias in the same way because the codon probability parameters do not enter the formulation of the Markov generator.

### Nonstationary and Time-Reversible Models Imply Different Conclusions regarding whether *ω* Is Constant between Lineages

As *ω* can be used to examine the difference between selection on different edges (Yang 1998; Huttley et al. 2000), we also tested how inference regarding *ω* on the human and mouse edges of the mammal data was affected by model choice.

**Fig. 7.—:**
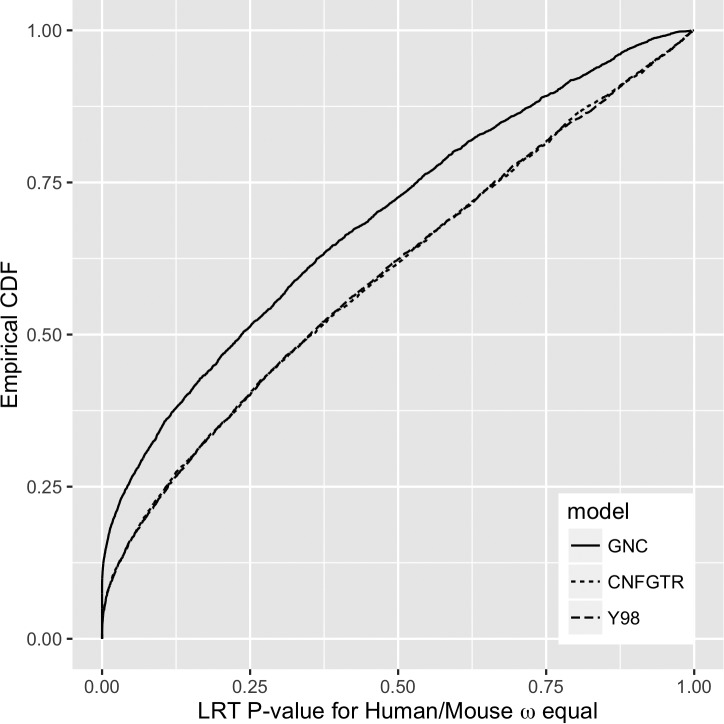
Empirical cumulative distribution functions of likelihood ratio test *P*-values between models with equal *ω* for mouse and human edges and unconstrained models based on Y98, CNFGTR, and GNC models over 4,039 alignments of human, mouse, and opossum protein coding genes.

The null hypothesis was that *ω* was equal between the human and mouse edges, against the alternative that it was not. We performed likelihood ratio tests (LRTs) on this basis. In all instances, no genetic distances were constrained to be equal. As for our other experiments, parameters other than *ω* and the scale parameter were equal across the tree for Y98 and CNFGTR and allowed to vary by edge for GNC. The results for the 4,039 mammals alignments are shown in figure 7. GNC indicated more violations of the null hypothesis, rejecting the null hypothesis at nominal 5% significance in 26.5% of cases. The two time-reversible models gave almost identical results, both rejecting the null in 16.4%.

## Discussion

We applied simulations, tests of model fit, and comparisons of statistical inference to examine some ways the assumption of time reversibility might mislead the scientist. Two representative time-reversible models were chosen for comparison (Y98 and CNFGTR), and a new, simple nonstationary model was developed (GNC). We tested three large sets of protein-coding sequences from mammals, vertebrates, and ants, and one large control set of intronic primate data.

We included a goodness-of-fit test that is capable of rejecting model fits if the model could not feasibly have generated the data. GNC performed the best of the three models. The ordering was consistent for every test we performed: GNC always tended to fit better than CNFGTR, which always outperformed Y98. In Kaehler et al. (2015), the equivalent experiment showed that a nonstationary nucleotide model was rejected at close to the type I error rate for one data set. Here the rejection rates were much higher (>74% for GNC in all cases). This shows that it is unlikely that any of the models considered here could feasibly have generated any of our data sets. We conclude that of the models considered, GNC tends to fit the data best, but that it may be possible to improve on GNC.

For every data set, genetic distances estimated under both time-reversible models were overestimated in comparison to those obtained from the nonstationary model. For the mammal and ant data sets, it was clear that this overestimation was proportional to a nonparametric measurement of nonstationarity, meaning that if the data supported time-reversibility, GNC tended to recapitulate the results obtained from CNFGTR or Y98. For the vertebrates, the signal was less clear, although there are some indications that this data set exists at the limits of our inference.

In conjunction with the results in Kaehler et al. (2015), we observe that overestimation of genetic distance by time-reversible models as proportional to nonstationarity is a consistent trend over data sets from vertebrates, invertebrates, and microbes for four different time-reversible nucleotide and codon models. Why fitting time-reversible models to data generated by nonstationary processes should lead to this phenomenon is an open question.

Next we addressed how inference about natural selection might be affected by the assumption of time-reversibility. We hypothesized that *ω* would be partially confounded with genetic distance, and found strong evidence for both time-reversible models and all data sets that when genetic distance was overestimated, *ω* tended to be underestimated. As overestimation of genetic distance is reduced when less nonstationarity is observed, so too is underestimation of *ω*, so again nothing is lost by using GNC rather than CNFGTR or Y98. Again, we observe that the effect is more pronounced in Y98 than CNFGTR, particularly for the ants.

In Yap et al. (2010), the conditional nucleotide frequency models of which CNFGTR is an example were introduced to combat bias that is observed in Y98 and other time-reversible codon models. We confirmed that GNC is also immune to such biases by conducting control experiments on intronic data from three primates. We perceive this advantage is a result of the simple form of the GNC parameterization. The expected result of the experiment was that *ω* should be approximately equal to one. This is what we observed for GNC, with Y98 again systematically underestimating *ω* in addition to being biased where GC content was not equal to 0.5. CNFGTR is interesting because it only slightly underestimates *ω*. This is in keeping with our other results if the amount of nonstationarity is low, as in that case we would expect *ω* to be only slightly underestimated.

To test the most important statistic that is estimated using codon models, we tested the hypothesis that *ω* is equal for the human and mouse edges in the mammal data. GNC was found to be more likely to reject the hypothesis that *ω* was equal and Y98 and CNFGTR, regardless of their apparent difference in specification and other experimental results here presented, gave almost indistinguishable results for this test. Time-reversibility seems to be the key distinction between the inference drawn from the models in this test. We also performed molecular clock tests similar to those in Kaehler et al. (2015) and showed that again time-reversibility caused more difference between inference than any other quality (see Supplementary Material online). We also confirmed trends noted in Kaehler et al. (2015).

Our tests of equality across lineages of genetic distance and natural selection show that these quantities clearly vary by lineage. It is therefore natural to assume that nonstationarity must vary from edge to edge as well. We have shown that the bias introduced by fitting time-reversible models to nonstationary data varies with nonstationarity. These biases cannot be a simple scaling of a phylogenetic tree, and so we speculate that inferences based on time-reversible models outside those analyzed in this work must also be susceptible to bias.

Understanding the extent to which the historical operation of natural selection has shaped the distribution of genetic variation has, to a very large extent, derived from application of codon models of sequence evolution. In the simplest sense, our ability to draw inference relies on how well these models represent the process of neutral sequence evolution. As we demonstrated previously (Kaehler et al. 2015), utilizing time-reversible nucleotide substitution processes distorts our estimation of the number of events in a manner that is proportional to the extent of nonstationarity. Nonstationarity is common across the tree of life (Karlin and Ladunga 1994). In this work, we have shown that the biases that were evident in time-reversible nucleotide models manifest in the codon case in such a manner as to underestimate the ratio of nonsynonymous to synonymous substitutions. As a consequence, the application of the time-reversible models to sequences with deep evolutionary divergence seems likely to give rise to estimates of natural selection that are artifactual, bringing into question conclusions regarding historical shifts in the operation of selection as have been recently reported (Sunagar and Moran 2015).

## Supplementary Material

Supplementary materials are available at *Genome Biology and Evolution* online.

## Supplementary Material

Supplementary Data

## Appendix

### Estimation of Selective Pressure under Nonstationary Models

We show that the definition of $K_{a}/K_{s}$ used in Goldman and Yang (1994) transfers unscathed to the context of nonstationary Markov models. Further, it is equivalent to the parameter *ω* in the following standard definition (Yang 2006, p. 59).

Define the instantaneous transition rates of a codon substitution process as

$$q_{ij}=\{\begin{array}{cc} 0, & \;\text{more}\;\text{than}\;\text{one}\;\text{difference}\;,\\ r_{ij}, & \;\text{one}\;\text{synonymous}\;\text{difference}\;\\ \omega r_{ij}, & \;\text{one}\;\text{nonsynonymous}\;\text{difference}\; \end{array}$$

where *r_ij_* is some representation of an evolutionarily neutral process and *ω* summarizes the influence of natural selection. Note that *r_ij_* is allowed to vary by transition in this formulation but has traditionally been further constrained to enforce, for instance, time-reversibility of the process. We denote the codon probability row vector π(t) for time t≥0.

Define two new transition matrices:

$$\begin{array}{c} q_{ij}^{S}=\{\begin{array}{cc} r_{ij}, & \;\text{one}\;\text{synonymous}\;\text{difference}\;,\\ 0, & \;\text{otherwise}\;, \end{array}\\ \text{and}\\ q_{ij}^{N}=\{\begin{array}{cc} r_{ij}, & \;\text{one}\;\text{nonsynonymous}\;\text{difference}\;,\\ 0, & \;\text{otherwise}\;, \end{array} \end{array}$$

so that $Q=\omega Q^{N}+Q^{S}$, where *Q*, $Q^{N}$, and $Q^{S}$ are the matrices comprised of *q_ij_*, $q_{ij}^{N}$, and $q_{ij}^{S}$, respectively, for their off-diagonal elements. Diagonal elements are calculated in the usual way for Markov generators.

Following Goldman and Yang (1994), we define *ρ_s_* and *ρ_a_* to be the rates of synonymous and nonsynonymous substitution per codon. They can be shown to be

$$\rho_{s}=-\pi (t)\text{diag}Q^{S}\;\text{and}\;\rho_{a}=-\omega \pi (t)\text{diag}Q^{N}.$$

Further, we define $\rho_{s}^{1}$ and $\rho_{a}^{1}$ as the potentials of synonymous and nonsynonymous substitutions when no selective constraints exist at the amino acid level, that is $\rho_{s}^{1}=-\pi (t)\text{diag}Q^{S}$ and $\rho_{a}^{1}=-\pi (t)\text{diag}Q^{N}$. These parameters correspond to $\rho_{s}^{\infty }$ and $\rho_{a}^{\infty }$ in Goldman and Yang (1994). Likewise, $3\rho_{s}^{1}$ and $3\rho_{a}^{1}$ represent the potentials per codon. As these have historically been termed the numbers of synonymous and nonsynonymous nucleotide sites per codon, we continue that terminology. The numbers of synonymous substitutions per synonymous site and nonsynonymous substitutions per nonsynonymous site are $K_{s}=\rho_{s}/3\rho_{s}^{1}=1/3$ and $K_{a}=\rho_{a}/3\rho_{a}^{1}=\omega /3$. It is therefore clear that

$$\omega =\frac{K_{a}}{K_{s}},$$

which shows that the usual definition and understanding of *ω* is sufficient in the context of nonstationary Markov models.
