# Supplementary material for: Standard Codon Substitution Models Overestimate Purifying Selection for Nonstationary Data
Source: Genome Biol Evol. 2017 Jan 5;9(1):134–49. doi: 10.1093/gbe/evw308 (PMC5381540; doi:10.1093/gbe/evw308)
Supplement: Supplementary Data [file evw308_Supp.pdf]

Supplementary Material for Kaehler, Yap & Huttley

Non-stationary and time-reversible models imply different conclusions regarding the molecular clock

To show that inferences drawn from the assumption of a non-stationary codon model differ to those from time-reversible models, we investigated how often the molecular clock hypothesis was violated for the mammals data set. We also performed this experiment to test the generality of results obtained in Kaehler et al. (2015) regarding the effect of using time-reversible models to test the molecular clock hypothesis. The molecular clock hypothesis in this context asserts that the genetic distance along the mouse and human edges should be identical. We expected that the molecular clock test would be rejected in the majority of cases (eg. Huttley et al., 2007).

We tested the hypothesis in two experiments where likelihood ratio tests (LRTs) were performed on 4,039 alignments. These tests are variants of the likelihood form of the relative rate tests established in Muse and Weir (1992). In all cases parameters other than the scale parameter and ω were constrained to be equal for all edges for the time-reversible models and allowed to vary by lineage for GNC and models were fitted on a node-rooted tree. In both experiments the genetic distance was also constrained to be equal on the human and mouse edges under the null hypothesis, and allowed to vary under the alternative. For the first experiment, ω was allowed to vary by edge. For the second, ω was constrained to be equal across all edges for the time-reversible models. The slightly unusual set-up for the latter was to enable comparison with the analogous test performed for Kaehler et al. (2015), which was performed without the complicating factor of including a selective pressure parameter.

The experimental setup for GNC was the same for both experiments and for this model the null hypothesis was rejected for 68.0% alignments at nominal 5% significance under asymptotic LRT assumptions. For the first experiment, for CNFGTR and Y98, 67.1% and 67.2% of alignments respectively rejected the null hypothesis at nominal 5% significance. For the second, the same respective proportions were 72.8% and 73.3%. All proportions are reported to one decimal place. The cumulative distributions of the LRT p-values are plotted in Figure S1. So where ω was allowed to vary by edge, the non-stationary model rejected the null hypothesis more often than the time-reversible models. Where ω was constrained for the time-reversible models, the opposite result was observed, confirming the result in Kaehler et al. (2015). In both cases, the results for the two time-reversible models were almost indistinguishable.

Figure S1: Molecular clock inference is affected by model time-reversibility and assumptions regarding ω. Empirical cumulative distribution functions of likelihood ratio test p-values between constrained clock-like and unconstrained models based on Y98, CNFGTR, and GNC models over 4,039 alignments of human, mouse, and opossum protein coding genes. For (a), ω was allowed to vary by edge in every case; for (b) ω was constrained to a single value for the tree for the time-reversible models.

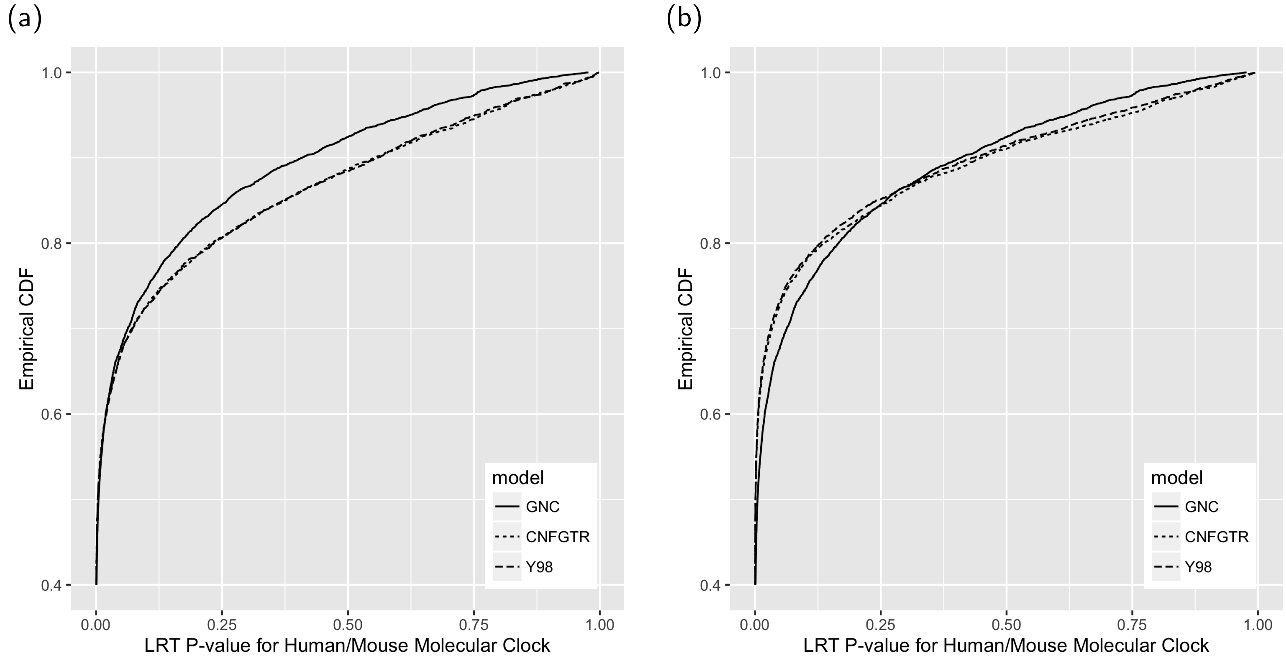

References

Huttley GA, Wakefield MJ, Easteal S. 2007. Rates of genome evolution and branching order from whole genome analysis. Molecular biology and evolution. 24(8):1722–1730.

Kaehler BD, Yap VB, Zhang R, Huttley GA. 2015. Genetic distance for a general non-stationary markov substitution process. Syst Biol. 64(2):281–293.

Muse S, Weir BS. 1992. Testing for equality of evolutionary rates. Genetics. 132(1):269–276.
